# System for the experimental evaluation of anthropomorphic hands. Application to a new 3D-printed prosthetic hand prototype

**DOI:** 10.1080/23335432.2017.1364666

**Published:** 2017-12-15

**Authors:** Immaculada Llop-Harillo, Antonio Pérez-González

**Affiliations:** a Grupo de Biomecánica y Ergonomía, Departamento de Ingeniería Mecánica y Construcción, Universitat Jaume I (UJI), Castellón, Spain

**Keywords:** Anthropomorphic hand, grasping evaluation, hand prostheses, design, 3D printing, finger coordination

## Abstract

In the present study, we propose a new actuation device and protocol for testing the grasping performance of low-cost 3D-printed hand prototypes. The actuation device is connected to the forearm of a healthy user and allows him to use his thumb and fingers to control any prototype moved by up to six tendons attached to this device. The protocol includes grasping actions on 24 different objects using eight typical grasp types to obtain a grasping ability score and information about the coordination of motion among the fingers. This study also presents a new design for a low-cost 3D-printed prosthetic hand, called the IMMA hand. Preliminary tests were performed with the IMMA hand and the actuation device on two subjects, using the protocol, to assess the validity of the device for the experimental evaluation of hand prototypes in early design stages. The analysis of the results of these tests shows that the coordination of motions among fingers is quite similar for both subjects, indicating a similar control of the artificial hand. Index and ring finger motions are highly correlated in over half of the grasp actions performed for both subjects.

## Introduction

The evaluation of grasping ability is essential to improve current anthropomorphic hand designs in fields like prosthetics and robotics. In the robotics community, many grasp quality metrics have been proposed in the literature to evaluate the grasp quality of manipulators. A recent survey by Roa and Suárez (2015) identified up to 24 different grasp quality metrics. These metrics play a key role in the analytical approach to the grasp-planning problem in robotics, often referred to as grasp synthesis, and also help quantify desirable properties like dexterity, force-closure, stability and equilibrium (Shimoga 1996; Sahbani et al. 2012). Comparison of the grasp metrics between artificial hands and the human hand has recently been proposed as a method to evaluate prosthetic hands (León et al. 2013).

However, the metrics cited above are analytical and focused on evaluating the stability of the object grasped in a particular grasping posture. They are mainly based on geometrical information such as the contact points and the contact normal between the hand and the object. These metrics do not evaluate the performance of the hand globally and they are obtained from simulations. Moreover, few analyses have been carried out to assess the ability of these hands to emulate the grasp modes of the human hand. In fact, evaluation of mechanical hands globally and not only for specific grasping postures has been dealt with only poorly in the literature. A recent empirical study by Belter et al. (2013) compared the characteristics of different prosthetic hands, but without defining outcome measures. The recent study by Feix et al. (2013) is, to our knowledge, the only such work that attempts to define an index which aims to compare the motion capability of robotic and prosthetic hands with that of the human hand, although it is somewhat limited, its computation being based only on the comparison of the position and orientation of the distal phalanges in different possible hand poses. Moreover, it is an analytical study, and important aspects for grasping such as friction, surface roughness and contact compliance are difficult to consider with analytical approaches.

Attention has also been paid to experimental approaches that aim to overcome the limitations observed with analytical approaches (Balasubramanian et al. 2012; Kim et al. 2012), considering effects such as friction, deformation, accuracy and sensitivity to positioning errors. In prosthetics and rehabilitation, several experimental protocols have been defined to evaluate the hand or the prosthetic hand function. Examples of these tests include the Sollerman Hand Function Test (Sollerman and Ejeskar 1995), the Southampton Hand Assessment Procedure (SHAP) (Light et al. 2002) and the Action Arm Research Test (McDonnell 2008). However, these tests are designed as clinical evaluation tools for application to subjects wearing the prosthesis or in the process of rehabilitation. The development of specific systems and protocols for the experimental evaluation of hand prototypes in the design phase is envisaged as a complementary tool to analytical metrics for improving anthropomorphic hand design.

Currently, however, the design of anthropomorphic hands is not based on sound grasp metrics or standard experimental protocols. Orthopaedic companies base their designs mainly on previous experience and user feedback, with an emphasis on grip modes and aesthetics. Developments in robotic hands like the DLR hand (Grebenstein et al. 2012) are moving towards a greater number of degrees of freedom (DOF), but their weight and size are necessarily greater than that of the human hand. Furthermore, price is not among the main factors considered in any of the previous approaches. More recent open designs of anthropomorphic hands, such as the Open Hand Project (2013), Flexy-Hand (Gyrobot 2014) and others, were compiled and analysed by the authors on a website (Biomechanics and Ergonomics 2016). These designs are moving towards low-cost models that enable self-manufacture using 3D printing technologies and customization.

Experimental tests and protocols for evaluating or comparing different mechanical hand designs globally in terms of their anthropomorphism or grasping ability and which are focused on improving the design of the artificial hands are scarce in the scientific literature. The development of simulation tools, benchmarks and protocols for evaluating hand designs numerically and experimentally is crucial for their future design. The prehensile ability of an artificial hand depends basically on three groups of factors: its mechanical design, the capacity of its actuators or motors and its control system. In this study, we assumed that it is desirable to decouple these three groups of factors to achieve an effective improvement of the design of artificial hands. Thus, in a first stage it is desirable to have a good understanding of the effect of the mechanical design of the hand and therefore it is also advisable that the actuation and the control of the hand prototypes are performed manually by a human operator. Accordingly, in the present study, we propose a new actuation device and protocol for testing the grasping ability of different hand prototypes experimentally. The actuation device has been designed to be placed on the forearm of a healthy subject, thus allowing him to use his thumb and fingers to control any hand prototype attached to this device moved by up to six tendons. The tendon-driven action is similar to that used in the human hand and intuitive for a human subject operating the hand. It also transmits some force-feedback information to the subject, which is very interesting to help achieve successful grasping. The use of a human controller eliminates the distorting effect that different control systems could have on the results when comparing different hands, thus allowing the hands to be compared only from the point of view of their mechanical design. If a human is in the loop actuating the device, we are close to having the best possible control (the human mind) and the best actuator for grasping (the human hand), although with the limitations imposed by the actuation device. Moreover, the actuation device designed in this study also permits the registration of the displacement of each driving cable during the tests, thereby allowing the coordination movements among the fingers to be studied. The information thus obtained can be used to consider the best option for the actuation of the artificial hand by motors, in a similar way to how it is performed by a human operator. The protocol to evaluate the hand prototypes includes grasping actions on 24 different objects using eight typical grasp types. In the present study, we also present a new design of a low-cost 3D-printed prosthetic hand with six DOF actuated by tendons, which was used to test the actuation device. Both the prosthetic hand and the actuation device are manufactured with 3D printing because this technology is typically used in low-cost hand prostheses and allows easy interchange of designs, which improves the comparability of the results. Preliminary tests were performed with this hand and the actuation device, using the proposed protocol, in order to assess the validity of the device for the experimental evaluation of hand prototypes in early design stages.

## Materials and methods

### Actuation device

A new device was designed that allowed a healthy subject to perform manual actuation of different low-cost 3D-printed models of artificial hands in order to test their grasping ability. Actuation by a healthy subject has the advantage that the control strategy for grasping is performed by the user’s brain, thus allowing different artificial hand prototypes to be compared from the point of view of the mechanical configuration and design, without the interference of different control strategies. The actuation device was designed taking into account the following specifications: (1) easy, fast, safe and secure attachment of the device to the forearm of a healthy adult subject; (2) adaptable to different hand-arm sizes of the users; (3) simple attachment of different artificial hands to the device must be possible and strong enough to resist a moment of 3 Nm; (4) intuitive and comfortable actuation of the artificial hand attached to the device (with up to six DOF) using tendons connected to the subject’s fingers and thumb; (5) the device must include sensors to measure and record the excursion of each actuating tendon during any grasping action; and (6) low weight and preferably manufactured by 3D printing technology.

**Figure 1.**
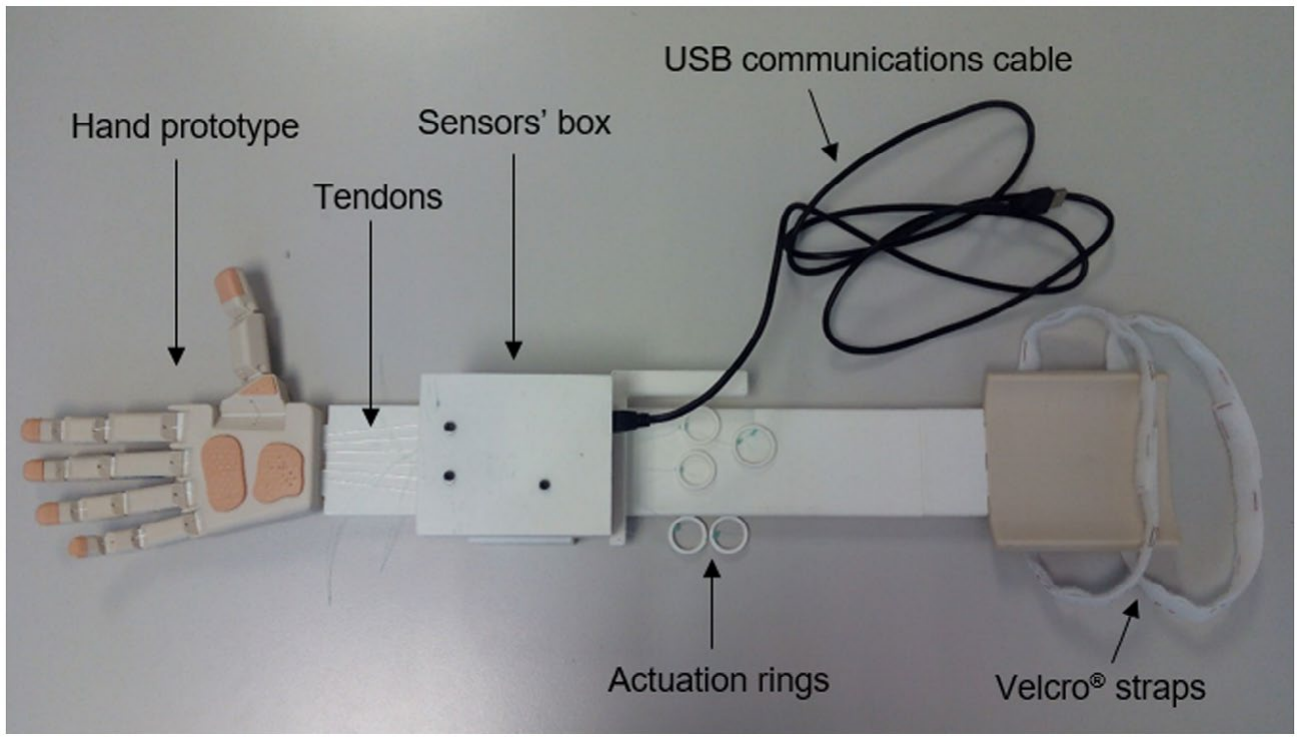
Actuation device with the IMMA hand prototype attached to it. The different components are shown: hand prototype, tendons, sensors box, actuation rings, USB communications cable and Velcro® straps for attachment to the forearm.

**Figure 2.**
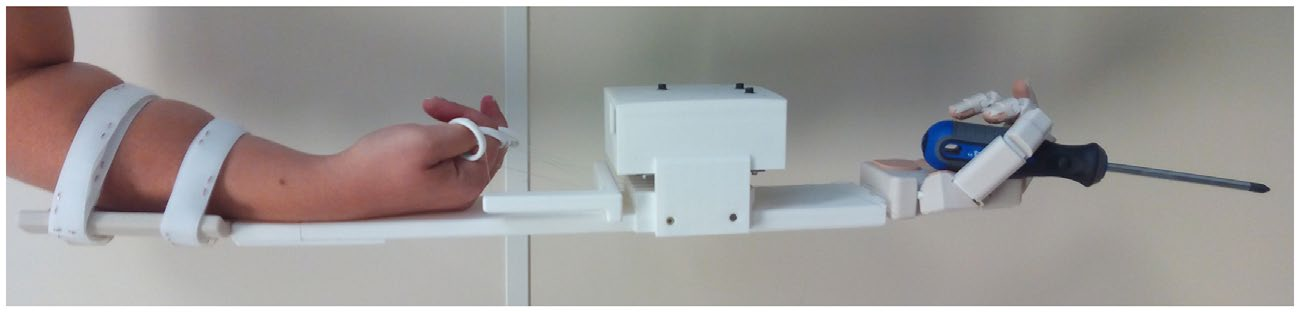
Device actuated by a subject during a grasping task.

Considering these specifications, several conceptual solutions were proposed for the device and preliminary prototypes were built to evaluate these conceptual solutions, mainly in reference to the method of actuation for the tendons. One of the options was to use a glove with external tendons whose excursion depended on the motion of the hand of the subject wearing this glove. The external tendons of the glove must be connected to the corresponding tendons of the artificial hand. This concept would permit an intuitive actuation of the artificial hand, but after building a first prototype this option was discarded because of the problems encountered when it came to obtaining a reliable solution for the attachment of the tendons to the glove and because of the difficulty involved in adapting a single glove size to the different hand sizes of the subjects who have to actuate the device, which affected the excursion of the tendons and hence the motion of the artificial hand. Finally, a concept based on the use of rings for the actuation of the tendons of the artificial hand was considered more reliable. Attachment of the device to the forearm of the healthy user was solved with a 3D-printed part and Velcro® straps, which allowed it to be attached in less than two minutes and to remain in place without feelings of pain or discomfort throughout the whole test. The actuation device was manufactured using 3D-printed technology. As mentioned above, this technology is common for low-cost 3D-printed hand prostheses and suitable for a low-weight solution. Moreover, it allows easy replication of the device by other research groups or developers, thereby promoting more accurate comparisons between hand designs. The connection of different artificial hands to the device is straightforward, requiring only the redesign of a customized connecting part at the end of the device. Our initial design is based on a press fit solution, which allows simple and sufficiently strong attachment of the low-cost prosthetic hand presented in this study. The solution adopted to measure the excursion of the tendons was the use of linear potentiometers connected to these tendons. These sensors allow the actuation of each finger to be compared during the tests and the analysis of synergies between the motion of the fingers, which can be used to reduce the number of motors for a prosthesis and simplify its control. An Arduino Uno board was used to collect the signal of the potentiometers and send it to a computer through a USB communication cable. This board was installed in a sensors box with the six linear potentiometers. The total weight of the actuation device including the sensors and the electronic board was 472 g. Figure 1 shows the prototype of the device that was designed, with a hand prototype attached to it. Figure 2 shows the actuation device, in a different view, worn by a healthy subject during a grasping task.

The actuation device allows the testing of hand prototypes actuated with up to six tendons by connecting these tendons to the corresponding rings of the device.

### Prosthetic hand

A new low-cost anthropomorphic prosthetic hand has been designed. The following specifications were considered for the design: (1) tendon-driven hand with six DOFs: independent flexion of the thumb and each finger and abduction motion of the thumb (the combination of flexion–extension and abduction–adduction movements of the thumb allows opposition of the thumb to orient the thumb distal phalanx to the distal phalanges of the fingers); (2) use of elastic elements to drive extension movements when the tendons are released; (3) natural rest position when not actuated; (4) simple and low-cost manufacturing and assembly, based on 3D printing technology; (5) main dimensions between the 50th percentiles of the male and female human hands; (6) friction coefficient and stiffness in the main contact areas of the palm and fingers similar to those of the human hand; (7) total weight no greater than that of the human hand; and (8) aesthetical appearance.

Some ideas were taken from previous developments to design a new prosthetic hand under these specifications. The design of the finger joints was inspired by the Flexy-Hand model (Gyrobot 2014), using elastic components, which connect the two phalanges of the corresponding joint (for interphalangeal joints) or the proximal phalange to the palm (for the metacarpophalangeal joints of the fingers and the carpometacarpal joint of the thumb). This solution simplifies the assembly of the hand and at the same time allows easy return to the rest position when the tendons are slack, without the need for additional springs. The orientation of the axes for the carpometacarpal joint of the thumb was taken as similar to that used in the ADA hand (Open Bionics 2016), but in our design the flexion and abduction movements of the thumb are actuated separately, as in the Tact hand (Slade et al. 2015) or the Dextrus hand (Open Hand Project 2013). The orientation of the fingers was taken as similar to that of the ADA hand (Open Bionics 2016) and the K1 hand (Keuster 2015).

Different materials based on different combinations of polylactic acid (PLA) and thermoplastic polyurethane (TPU) were used in the construction of the hand to obtain the proper stiffness and friction coefficient in each area in order to improve the grasping ability: PLA SOFT-Flexible (mixture of PLA and TPU using hexamethylene diisocyanate) was used for the palm and phalanges; NinjaFlex® (special formulation of TPU with high flexibility and durability) was used for the elastic joints; FilaFlex® (based on TPU with additives) was used for inserts located in the finger pads and some areas of the palm, which are the parts of the hand with more contact with the objects, because of its good compliance and greater friction coefficient. Different tests were performed using a dynamometer to characterize the mechanical behaviour of the different parts of the hand and validate the specifications. To obtain the approximate contact stiffness, we used a cylindrical indentor with a flat end (3.8 mm in diameter) following a procedure similar to that described in a previous work (Pérez-González et al. 2013). The mean stiffness obtained for the different parts of the hand was, respectively, 5.1 N/mm for FilaFlex® inserts used in the distal phalanges, 13.9 N/mm for the main body of the hand and phalanges made of PLA SOFT-Flexible and 6.3 N/mm for the FilaFlex® inserts used in the palm. The rotational stiffness of the joints were obtained by measuring the force necessary to rotate the joint until close to its limit and dividing the torque applied by the angle rotated. The stiffness obtained for the finger joints was dependent on the joint width, with an average value of 1.8 N mm/rad per mm of width of the joint. The friction coefficient of the FilaFlex® inserts and that of the PLA SOFT-Flexible surfaces of the hand against aluminium were obtained by measuring the force needed to slide a prismatic block of this material in contact with the corresponding part of the hand. An approximate friction coefficient of 0.63 was obtained for the FilaFlex® inserts, and 0.22 in the case of the PLA SOFT-Flexible.

**Figure 3.**
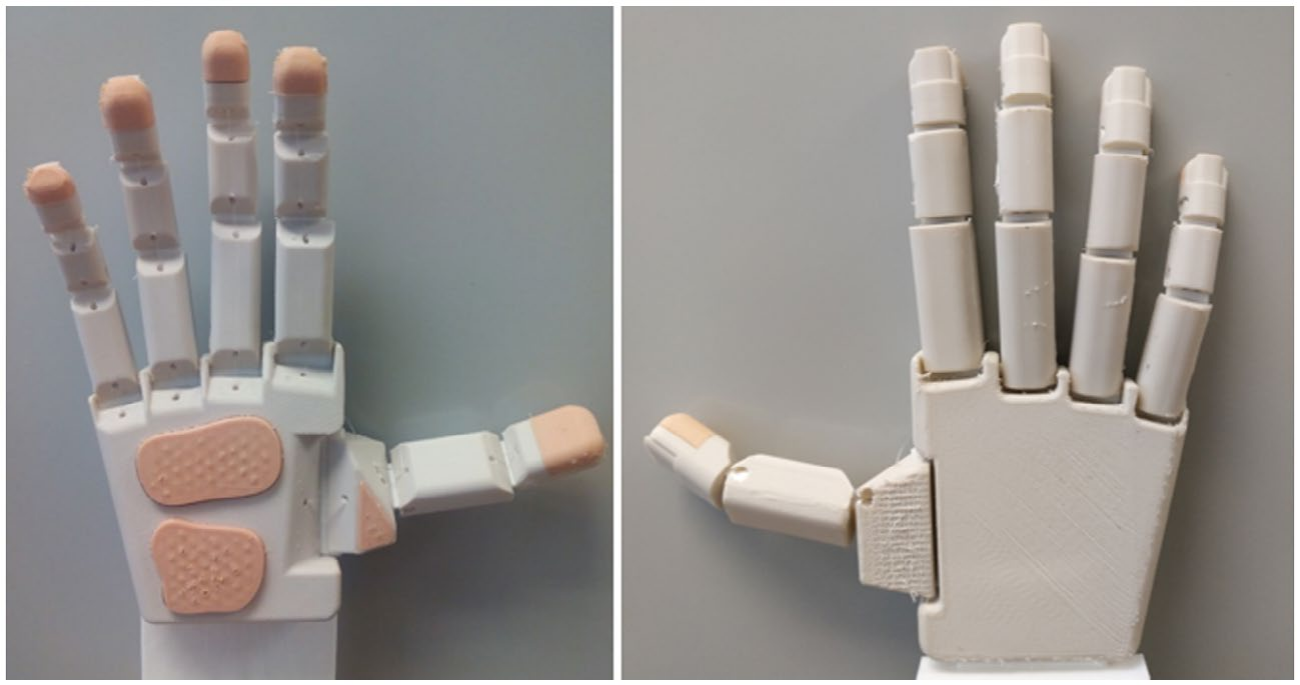
Prototype of the IMMA hand.

Four nylon fishing lines, with a diameter of 0.5 mm and a strength of 135 N, were used as tendons for flexing the fingers and two more for flexing and abducting the thumb, with an appropriate routing through the phalanges. A knot was used to attach the tendon to the distal phalanges, this knot being hidden below the inserts used in the distal phalanges to improve the aesthetics. The dimensions of the fingers and palm of the hand were selected so to be between the 50th percentiles of human male and female hands, based on data obtained in the authors’ research group, with a hand length (from the most proximal palmar point to the tip of the middle finger) of 184.4 mm and a hand width (at the metacarpal heads) of 80 mm. The total weight of the hand was 131.5 g and the cost of its 3D-printing material was less than 10 €. The prototype of the hand, referred to as the IMMA hand, is shown in Figure 3.

### Protocol for testing hand prototypes

A standard protocol for testing hand prototypes would help obtain experimental evidence with which to compare the grasping performance that can be reached with different hand designs. The use of the actuation device developed in the present study, connected to different hand prototypes, allows straightforward comparison of different hands. As a healthy operator is used to actuate the hand prototypes with this device by pulling the rings, the control issues are avoided and only the mechanical aspects of the design (materials, geometry, friction coefficients, mechanical advantage, efficiency, etc.) are considered in the evaluation and comparison of the artificial hands.

**Figure 4.**
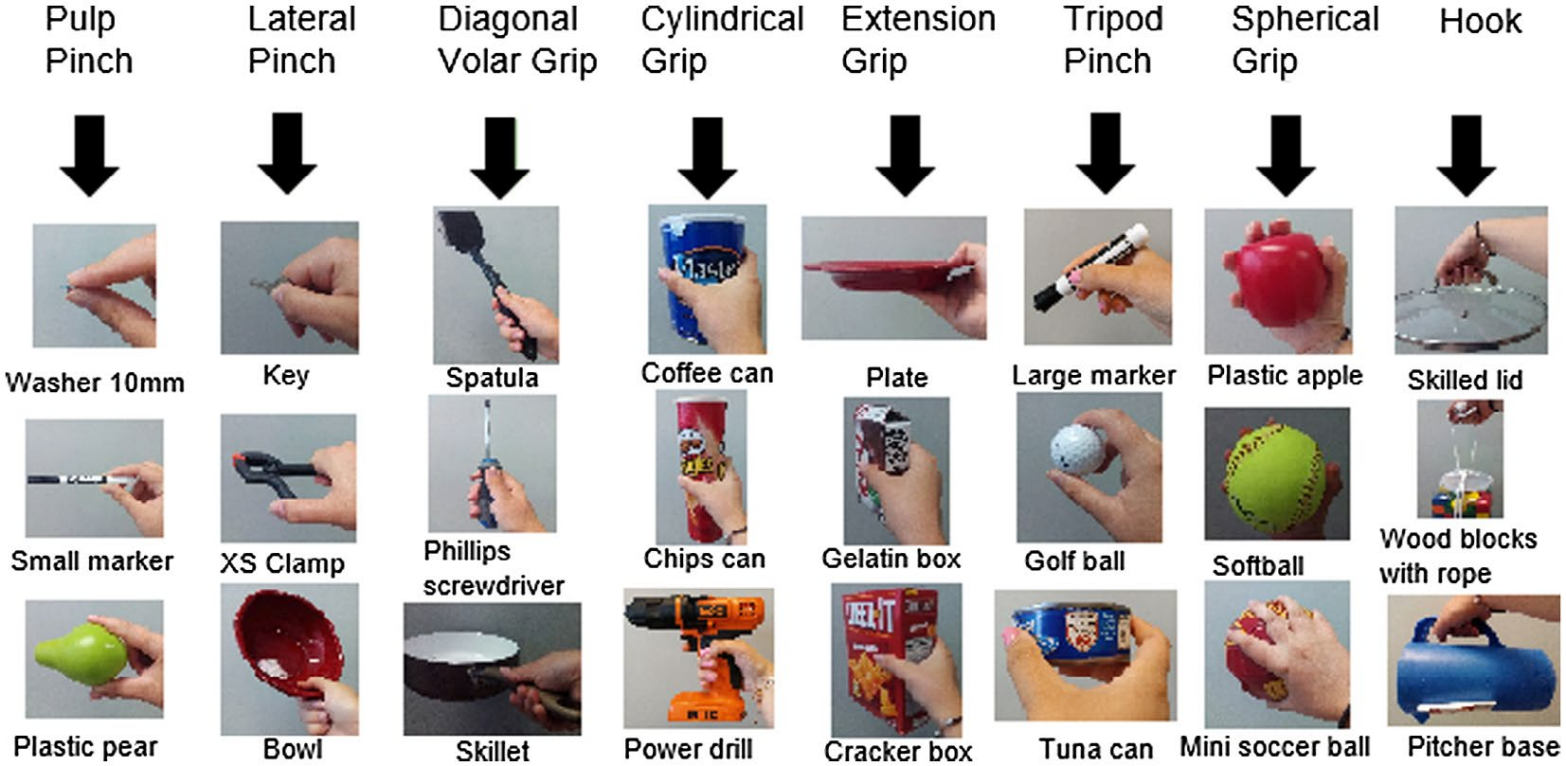
Grasping types and objects from the YCB set used in the protocol.

In order to establish the protocol, a group of objects and grasping tasks must be selected. We decided to choose the objects from a standard set of objects recently proposed for establishing benchmarks in manipulation research, the Yale-CMU-Berkeley Object and Model Set (Calli et al. 2015), because this set has been widely distributed among the robotics and biomechanics research community. The set is composed of 73 different objects including some elements typically found in activities of daily living (ADL). From this set, we selected a subset of 24 objects, divided into eight groups of three objects, with each group corresponding to objects typically grasped with one of the eight different grasping postures or grasp types (GT). We partially based the selection of these eight GT on the results of a previous field study conducted in the authors’ research group about grasps used in ADL (Vergara et al. 2014) and on previous research by other authors in the area of rehabilitation and prosthetics (Sollerman and Ejeskar 1995; Light et al. 2002). Figure 4 shows the eight different GT considered (pulp pinch, lateral pinch, diagonal volar grip, cylindrical grip, extension grip, tripod pinch, spherical grip and hook grip) and the three objects from the YCB set used for each of them. The three objects for each GT were selected with a view to varying the size, shape and weight. For the hook grasp, a combination of two objects in the set, rope and coloured wood blocks, were selected as one of the objects, the rope being used as a handle for lifting the wood blocks container.

**Figure 5.**
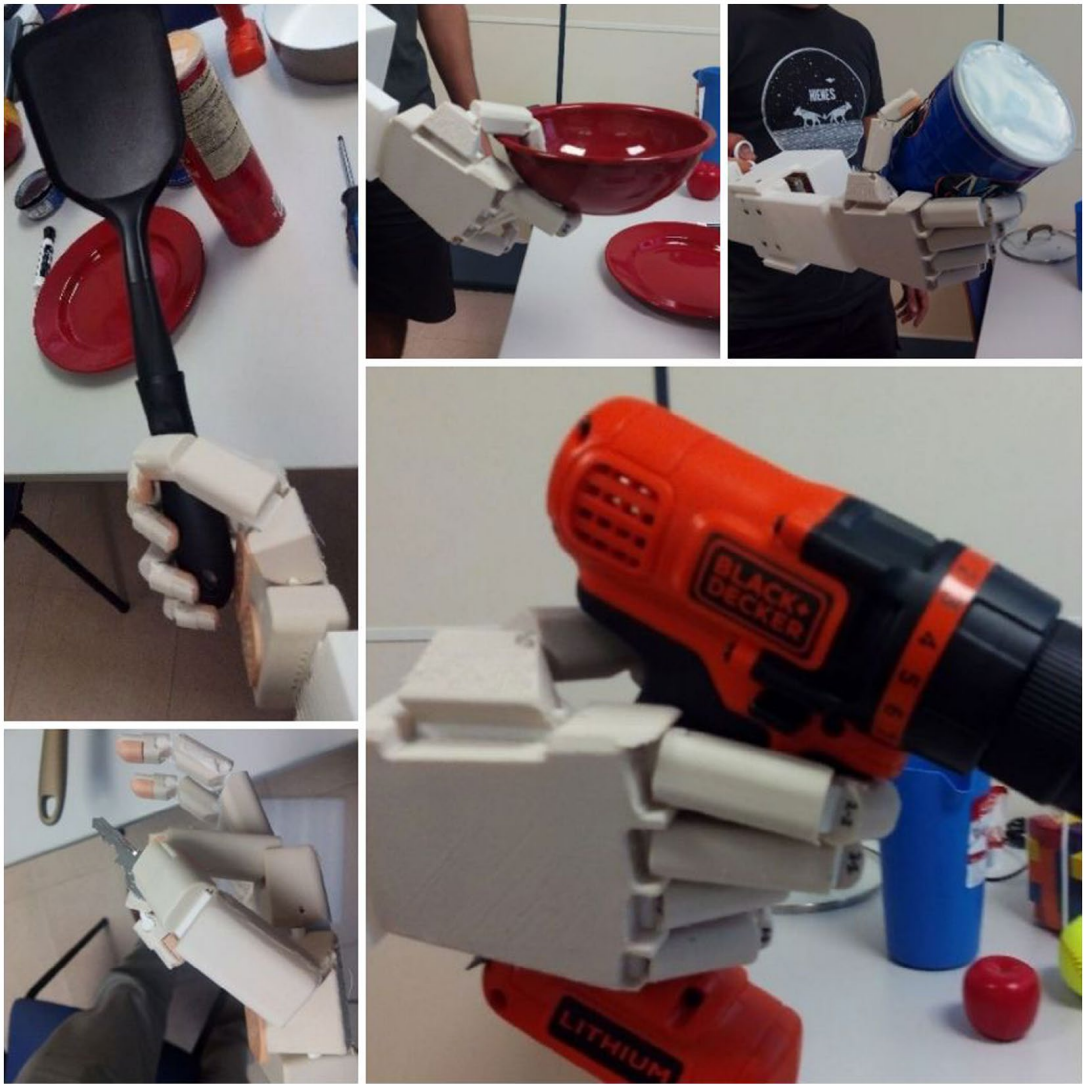
Examples of grasps performed by a subject wearing the actuation device with the IMMA hand.

For the test, the healthy subject, wearing the actuation device with the hand prototype attached to it, was instructed to grasp different objects for close to three seconds. The subject was in a standing position during the test and the test operator held the objects close to the artificial hand, in the correct orientation for performing the desired GT. The subject was instructed to try to use, as much as possible, the GT corresponding to each object. During the test, the operator registered the success or failure to keep the grasp without the object falling and scored the results, assigning 1 point if the grasp was completed successfully in the first trial, 0.6 points if completed successfully in the second trial, 0.4 if completed in the third trial, 0.2 points if completed with a grasping posture other than the one specified and 0 points if the grasp was unsuccessful. Scores for the three objects with each grasping posture were added to obtain the final score for this grasping posture. Scores for all the objects/tasks were then added to obtain the final score for the artificial hand. Normalized scores can be obtained by dividing by the maximum possible scores. The electronics of the actuation device also registered the excursion of the tendons during the test duration as well as the time spent on the task. Figure 5 shows some examples of task executions with different objects included in the protocol.

### Preliminary tests

A preliminary experiment was undertaken with the aim of testing the proposed protocol, the performance of the actuation device and the performance of the IMMA hand. Two adult male subjects, members of the research group (age 50 and 43, hand length 18.5  and 20.0 cm, hand width 9.5  and 9.0 cm, respectively), without any previous hand pathology, participated in the experiment, which was approved by the Ethics Committee of the university. They followed the protocol as described in the previous section, performing the grasping actions with the 24 objects in random order, in the same session. The excursion of the actuation tendons registered by the electronic box of the device during the tests was analysed with the aim of finding correlations or synergies among the different actuating tendons. For this analysis, only the motion of the tendons from the extended position to the final grasping posture was considered, but not the return to the extended position, which should be performed by the elastic joints because this is the relevant portion of the motion to analyse the possible effect of the synergies on the reduction of the number of actuators for the artificial hand. For the purposes of the analysis, a correlation coefficient between two tendons higher than a threshold of 0.9 was taken as high enough (within the limit of the possible experimental errors) to consider the possibility of using the same actuator for two different tendons.

## Results

The preliminary tests confirmed the validity of the actuation device for actuating the IMMA hand prototype. The device was easily attached to the forearm of both subjects in less than two minutes and the hand prototype was attached to the device simply with the press fit, which could resist the moment generated when lifting the heaviest object in the set (around 3 Nm). The connection of the tendons to the electronics box took around 30 min, and was the slowest action in this attachment process. Both subjects found it easy to control the artificial hand using the rings connected to their own fingers, although they found it somewhat difficult to control the thumb abduction. Even though the device was light, some fatigue was reported by the subjects at the end of the test.

Table 1 shows the objects for which each of the two subjects failed in the grasp, grouped by GT. Pulp pinch was the most difficult GT, followed by spherical grip. All the objects corresponding to lateral pinch, diagonal volar grip, cylindrical grip, tripod pinch and hook grip were grasped successfully by both subjects. Following the scoring method proposed in the protocol, the grasping ability obtained by the IMMA hand was 71% with the first subject and 75% with the second subject.

**Table 1.** Objects from the YCB set grouped by grasp type, for which any of the subjects failed in the grasping task.

Grasp type (GT)	Subject 1	Subject 2
Pulp pinch	Plastic pear^a^, small marker, washer	Plastic pear^a^, small marker, washer
Extension grip	Plate	–
Spherical grip	Softball, mini soccer ball	Softball, mini soccer ball

a The object was grasped but the GT used was considered a lateral pinch.

**Figure 6.**
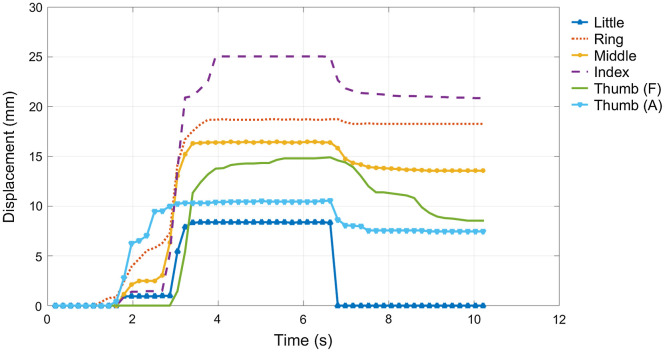
Example of excursion of the tendons during the grasp of the power drill with the IMMA hand operated by Subject 2 using the actuation device.

Figure 6 shows an example of the evolution of the excursions for the six tendons used in the anthropomorphic hand. An excursion of 0 mm corresponds to the rest position with the hand extended by the flexible joints. The plateau in the central part of the curves corresponds to the stable grasp posture maintained around three seconds. The final part of the curves after releasing the object indicates that the hand prosthesis did not recover the initial extended posture autonomously because the extending moment introduced by the flexible joints of the IMMA hand is unable to overcome the friction force existing in the linear potentiometers of the actuation device.

Table 2 shows the frequency of cases, among the successful grasps for each subject, for which the correlation coefficient between the excursions of each pair of tendons was higher than 0.9. The most frequently correlated motions corresponded to those of the index and ring fingers, for both subjects, followed by those of the middle and ring fingers. The correlation between both tendons (flexion and abduction) moving the thumb is also significant. The ring finger is the one presenting the highest motion correlation with other fingers. Thumb flexion is highly correlated for nearly one-third of the cases with index finger flexion and nearly one-quarter of the cases with ring finger flexion, the correlation with middle finger flexion not being frequent.

**Table 2.**
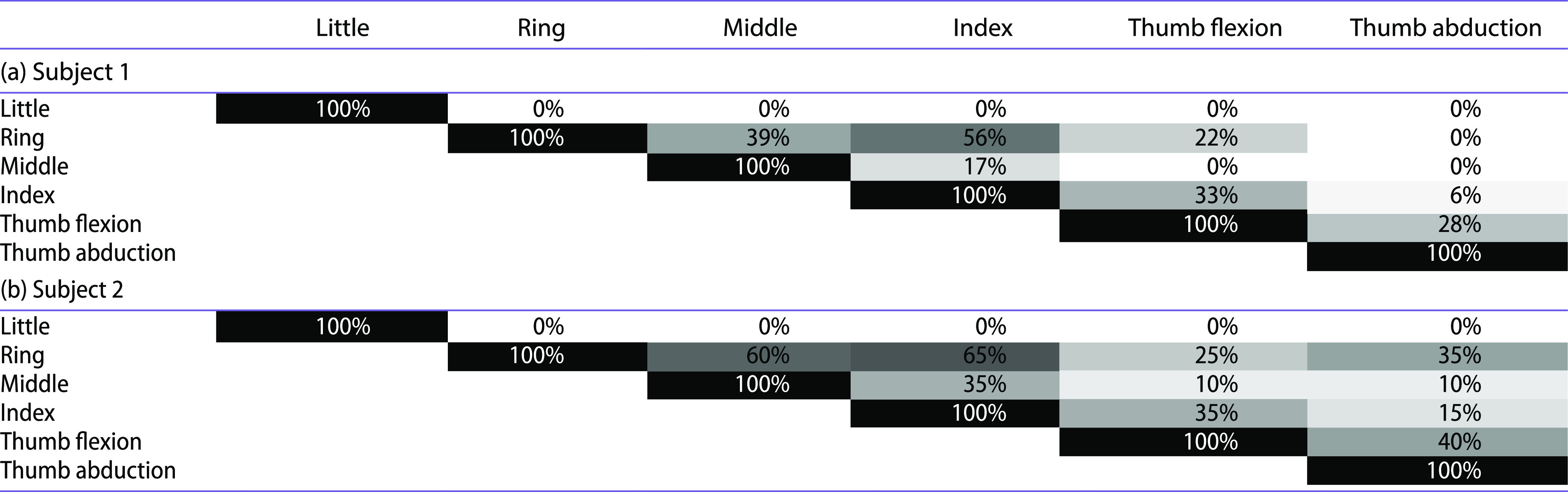
Frequency of cases, among successful grasps for each subject, for which the correlation coefficient between the excursions of each pair of tendons was higher than 0.9 for (a) Subject 1 and (b) Subject 2. Darker colours correspond to higher correlations.

## Discussion

In this study, we have presented a new actuation device, which can be used for the experimental evaluation of artificial hands. This evaluation can be employed to assess the goodness of other analytical metrics to give an index of the grasping capabilities of an artificial hand design, but can also provide complementary information in early design stages, which is helpful for improving the design. The device can be actuated by a healthy subject in order to move the fingers and thumb of the artificial hand, through cables connected to up to six tendons of the artificial hand. As the control of the hand through the device is carried out by the human operator, the possible differences in control methods for the artificial hand are not included in the evaluation. Moreover, manual actuation is helpful to provide some feedback about the force exerted on the object. To our knowledge, this is the first development of a device to manually actuate artificial hand prototypes for assessing their grasping capabilities. Other studies used previously instrumented gloves or other devices for teleoperating robotic hands (Farry et al. 1996; Xu and Todorov 2016), but in these methods the artificial hand is moved with motors and the devices used for teleoperation were used only to send signals to actuate the motors of the hand. Our solution of manual operation has the advantage of offering good feedback to the user in order to improve control while grasping. Additionally, the system provides information about the actuation coordination performed in the different DOFs of the artificial hand to grasp each object, which can be very useful for designing under-actuated hands, because those DOFs actuated in a very coordinated manner for most of the objects are candidates for being actuated with the same motor.

From the preliminary tests, some limitations have been observed in the current design of the actuation device. The friction introduced by the potentiometers of the device should be reduced because it prevents the extension of the fingers of the IMMA hand when the tendons are released. However, this fact does not breach any of the specifications proposed for the hand because it is able to return easily to the rest position when the tendons are slack if the hand is not connected to the actuation device. Moreover, this limitation does not invalidate the results of the experiments because only the motion from the initial extended position to the grasp posture is considered for the analysis of the correlations between the actuating tendons. The following improvements are envisaged for future versions of the device:

• A redesign of the device for a configuration in parallel to the arm of the user instead of as a prolongation of it, thereby allowing a final position of the artificial hand located at the same distance from the body as the user’s hand but displaced towards the sagittal plane. This improvement would allow a more natural and ergonomic actuation of the artificial hand, especially for grasping objects resting on a table, and would also reduce the moment transferred by the device to the forearm when grasping heavy objects.
• A more comfortable and ergonomic method for connecting the cables to the user’s fingers, especially for the thumb, and adaptable to different sizes of the user’s hand.
• A change in the method of measurement of the excursions of the tendons to reduce the friction introduced by the linear potentiometers on the tendons.
• The use of wireless connection between the device and the computer to improve the portability of the device.

A protocol has been proposed for the experimental testing of artificial hands for use in robotics or prosthetics. The protocol is based on the manual actuation of the artificial hand with the actuation device in order to undertake grasping actions with different objects and grasp types. Three different objects from the YCB set (Calli et al. 2015) were selected for each of eight different GT, all of them characteristic of the main grasping postures used with the human hand. With this protocol, we are able to obtain a score for the grasping ability of each prosthetic hand as well as information about the coordination of motion among the fingers that can be used for further analysis of the motors required to actuate the prosthesis and its control strategy. The protocol is centred on grasping actions, although it could be improved in the future to include other non-grasping postures, such as point and platform, which a multi-grasp prosthetic hand should also be capable of achieving according to previous studies (Balasubramanian and Santos 2014).

A new design for a low-cost 3D-printed prosthetic hand, called the IMMA hand (2017), has been presented in this study. The hand has six degrees of freedom, two of them for the thumb to allow opposition, actuated by tendons, and combines different materials to obtain an appropriate friction coefficient and compliance in the contact areas. The stiffness obtained in the finger and palm inserts is comparable to that measured in the distal phalanges of the fingers (Pérez-González et al. 2013). Moreover, a similar friction coefficient to that observed in the human hand (O’Meara and Smith 2001) was obtained between the IMMA hand inserts and aluminium.

The preliminary tests performed with the actuation device indicate a fairly good grasping capability, except for pulp pinch and spherical grip. A change in the orientation of the thumb joints could improve these limitations and is going to be analysed in the next version of this hand design.

The analysis of the results of the preliminary tests performed on two subjects shows that the coordination of motions among fingers is quite similar for both subjects (Table 2), indicating a similar control of the artificial hand by both subjects. Index and ring finger motions are highly correlated in more than half of the grasp actions performed, thumb flexion being more correlated with these two fingers than with the middle finger. A recent study about coordination of motion among the joints of the human hand in dexterity tests and ADL has observed the highest correlations between index and middle metacarpophalangeal joints (Gonzalez-Sanchez et al. 2016). Our results also show significant coordination between the flexion of the index and middle fingers, but a higher coordination has been observed between the ring and index fingers, although the experiments are not totally comparable. Thumb flexion and abduction are correlated for near 30%-40% of the grasping tasks, but this correlation can be due to the fact that both motions are actuated with the same ring attached to the subject’s thumb. This particularity of the actuation device is considered a limitation that makes the control of the thumb of the artificial hand difficult and should be improved for further developments of this device.

## Conclusion

We have proposed a framework for the experimental evaluation of robotic and prosthetic hands in order to assess their grasping capabilities when controlled by a human operator. The actuation device developed can be used easily by healthy users to test the artificial hands, providing information about the control strategy employed during grasping because it registers the excursion of the actuating tendons during the grasp action. The control strategy followed by different users has been shown to be similar in a preliminary test with two users. Future work will focus on improving the design of the actuation device to make it more ergonomic and on testing and comparing different artificial hands using this framework to obtain conclusions for improving future designs of low-cost prosthetic hands.

## Disclosure statement

No potential conflict of interest was reported by the authors.

## Funding

This work was supported by the Spanish Ministry of Economy and Competitiveness and ESF [grant number BES-2015-076005]; Spanish Ministry of Economy and Competitiveness and ERDF [grant number DPI2014-60635-R].
